# Sensitive bispecific chimeric T cell receptors for cancer therapy

**DOI:** 10.21203/rs.3.rs-4253777/v1

**Published:** 2024-04-22

**Authors:** Sylvain Simon, Grace Bugos, Rachel Prins, Anusha Rajan, Arulmozhi Palani, Kersten Heyer, Andrew Stevens, Longhui Zeng, Kirsten Thompson, Jason P. Price, Mitchell K. Kluesner, Carla Jaeger-Ruckstuhl, Tamer B. Shabaneh, James M. Olson, Xiaolei Su, Stanley R. Riddell

**Affiliations:** 1 Translational Sciences and Therapeutics Division, Fred Hutchinson Cancer Center, Seattle, WA 98109, USA.; 2 Department of Immunology, University of Washington, Seattle, WA 98195, USA.; 3 Department of Cell Biology, Yale School of Medicine, New Haven, CT 06520, USA.; 4 Yale Cancer Center, Yale University, New Haven, CT 06520, USA.; 5 Department of Biochemistry, University of Washington, Seattle, WA 98195, USA; 6 Seattle Children’s Research Institute, Ben Towne Center For Childhood Cancer Research, Seattle, WA 98105, USA.; 7 Human Biology Division, Fred Hutchinson Cancer Center, Seattle, WA 98109, USA; 8 Department of Medicine, University of Washington, Seattle, WA 98195, USA

## Abstract

The expression of a synthetic chimeric antigen receptor (CAR) to redirect antigen specificity of T cells is transforming the treatment of hematological malignancies and autoimmune diseases [1–7]. In cancer, durable efficacy is frequently limited by the escape of tumors that express low levels or lack the target antigen [8–12]. These clinical results emphasize the need for immune receptors that combine high sensitivity and multispecificity to improve outcomes. Current mono- and bispecific CARs do not faithfully recapitulate T cell receptor (TCR) function and require high antigen levels on tumor cells for recognition [13–17]. Here, we describe a novel synthetic chimeric TCR (ChTCR) that exhibits superior antigen sensitivity and is readily adapted for bispecific targeting. Bispecific ChTCRs mimic TCR structure, form classical immune synapses, and exhibit TCR-like proximal signaling. T cells expressing Bi-ChTCRs more effectively eliminated tumors with heterogeneous antigen expression in vivo compared to T cells expressing optimized bispecific CARs. The Bi-ChTCR architecture is resilient and can be designed to target multiple B cell lineage and multiple myeloma antigens. Our findings identify a broadly applicable approach for engineering T cells to target hematologic malignancies with heterogeneous antigen expression, thereby overcoming the most frequent mechanism of relapse after current CAR T therapies.

The adoptive transfer of T cells expressing a chimeric antigen receptor (CAR T) specific for B-cell lineage antigens such as CD19 and TNFRSF17 (BCMA) can induce rapid regression of relapsed or refractory lymphoma, acute leukemia and multiple myeloma [1–6]. Despite high initial response rates, a majority of patients relapse after CAR T treatment [2–6]. Heterogeneity in the expression level or complete loss of the target antigen on the tumor have been identified as frequent causes of relapse [8–10]. Genomic analysis of tumors identified preexisting or emergent clones that escape CAR T cell recognition as a result of bi-allelic loss of the gene encoding the target antigen, point mutations in the epitope, or downregulation of antigen expression due to epigenetic silencing of promoter or enhancer regions [11, 18]. These findings illustrate the need for T cell therapies that provide both multi-specific and highly sensitive antigen recognition.

CAR design was inspired by the understanding that two signals mediated by the T cell receptor (TCR) and a costimulatory molecule were required for full T cell activation [19]. Each TCR assembles with CD3δ, CD3ε, CD3γ, and CD3ζ homo- or heterodimers providing 10 immune-receptor-tyrosine-based-activation-motifs (ITAMs) for modification upon antigen recognition [20, 21]. The earliest CARs fused a single chain variable fragment (scFv) of a monoclonal antibody to the CD3ζ subunit, which has only three ITAMs [22]. Costimulation was added by incorporating the signaling domain of one or more costimulatory molecules into the receptor architecture, rather than engagement of separate molecule as occurs with TCR recognition [23–26]. Differences between CARs and TCRs are evident by comparison of signaling after engaging antigen. CAR T cells exhibit reduced phosphorylation of ZAP-70 and LAT signaling intermediates compared to a TCR [13–15], which may contribute to their markedly reduced antigen sensitivity [15–17, 27]. To improve sensitivity, receptors that better engage the TCR apparatus have been designed [28–31]. The HLA independent T cell receptor (HIT) and the synthetic T cell and antigen receptor (STAR) fuse the VH and VL chains of an antibody directly to TCR constant alpha (TRAC) and beta (TRBC) chains respectively [30, 31]. To reduce mispairing with endogenous TCR chains, the HIT receptor transgene was inserted into the TRAC locus, and the STAR receptor fused VH and VL chains with murine TCR constant chains that contained an interchain disulfide bond to promote pairing [30, 31]. Although more sensitive than conventional CARs, HIT and STAR receptors recognize only one antigen and would not prevent the outgrowth of antigen negative tumors.

We sought to develop a synthetic chimeric T cell receptor (ChTCR) that could target two antigens with high sensitivity. We designed a new construct termed the “full ChTCR” where the tumor-targeting scFv, rather than a VH or VL fragment, is fused to TRAC chain while the TRBC chain is left void of an antigen binding domain. The full ChTCR was superior to the split ChTCR format used in HIT and STAR receptors and was readily adaptable for bispecific antigen targeting by linking a second scFV specific for a different tumor antigen to the TRBC chain. Compared to monospecific ChTCRs and bispecific CARs, bispecific ChTCRs (Bi-ChTCRs) were superior at recognizing and eliminating heterogeneous and antigen low tumor cells in vitro and in vivo. This new Bi-ChTCR design can be broadly applied for sensitive targeting of multiple pairs of lineage antigens on B cell malignancies and multiple myeloma demonstrating significant promise for rapid translation to clinical application.

## Chimeric TCRs recapitulate TCR structure, synapse formation and signaling

**Figure 1.**
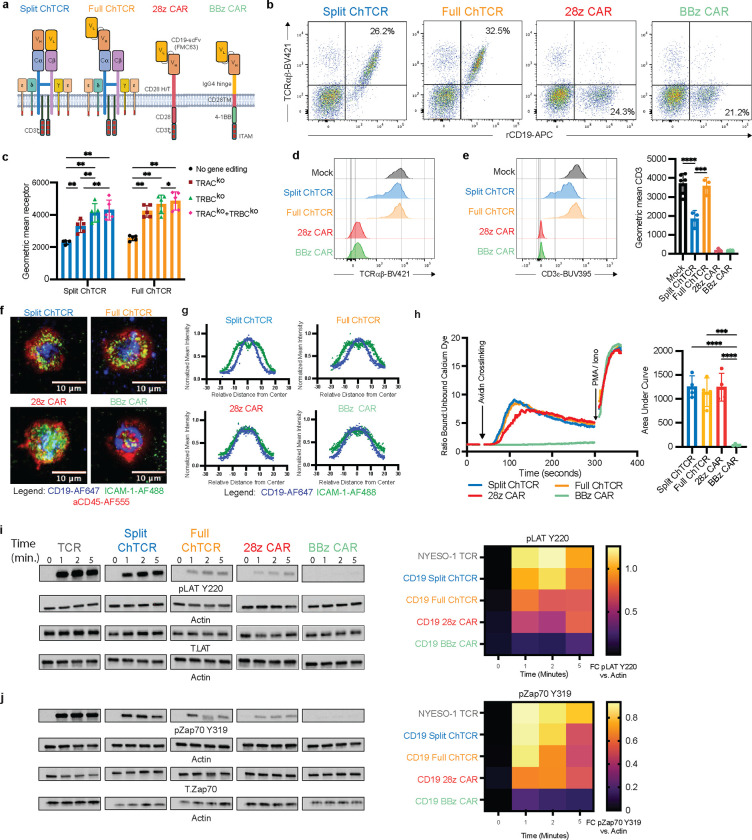
Chimeric TCRs expressed in T cells reproduce canonical TCR structure, synapse formation and proximal signaling **a.** Schematic of CD19-specific ChTCRs and CARs: Left to right - Split ChTCR V_H_C_α_: FMC63 variable heavy chain-TCR alpha constant chain, V_L_C_β_: FMC63 variable light chain-TCR beta constant chain; Full ChTCR: FMC63V_L_V_H_-C_α_:C_β_ TCR. Both ChTCRs are shown in association with endogenous CD3εδ, CD3ζζ and CD3γε subunits. CD28ζ CAR with FMC63 V_L_V_H_ scFv linked to CD28 hinge/transmembrane and co-stimulatory domain, and CD3ζ; BBζ CAR with FMC63 V_L_V_H_ scFv linked to IgG4 hinge, CD28 transmembrane domain, 4–1BB co-stimulatory domain and CD3ζ. **b.** Representative flow plots of primary CD8 T cells stained with anti-TCRαβ antibody and recombinant CD19 protein after lentiviral transduction and base editing to knock-out endogenous TCRαβ expression. **c.** Geometric mean of rCD19-APC binding to primary T cells transduced with Split or Full ChTCRs with and without base editing of TCRα, TCRβ or both TCRαβ chains (n=5 biological independent samples). *P<0.05, **P< 0.01 using two-way ANOVA. **d.** Representative TCRαβ expression by mock unedited and TCRαβ gene edited ChTCR^+^ and CAR^+^ T cells. **e.** Left: Representative CD3ε expression by mock unedited and TCRαβ gene edited ChTCR^+^ and CAR^+^ T cells. Right: geometric mean ± SD of CD3ε-BUV395 fluorescence (n=4 independent donors). *** P< 0.001, **** P< 0.0001 by two-way ANOVA. **f.** Left: Representative TIRF microscopy images of ChTCR^+^ and CAR^+^ T cells interacting with a soluble lipid bilayer functionalized with ICAM-1 extracellular domain (green), CD19 extracellular domain (blue) and stained with an anti-CD45-AF555 antibody. Scale bars = 10μm. **g.** Normalized mean intensity of ICAM-1-AF488 (green) and CD19-AF647 (blue) staining across cell radiuses in synapses; dots represent mean at each position with solid trend line, n=100 cells. **h.** Left: Representative calcium flux measured after antigen crosslinking of T cells expressing each of the specified receptors. The y-axis shows the ratio of calcium bound to unbound Indo-1 dye over time on the x-axis. Arrows indicate crosslinking of receptors and addition of PMA/ionomycin. Right: Area under the curve of calcium flux measurements over 300 seconds after antigen cross-linking, (n=4 independent experiments). *** P< 0.001, **** P< 0.0001 by two-way ANOVA. **i.** Left: Representative western blot of LAT pTyr^220^, Actin and LAT after antigen activation of T cells expressing each of the indicated receptors. Right: heat map of mean band intensity of LAT pTyr^220^ normalized to actin loading control (n=3 independent experiments). **j.** Left Representative Western Blot analysis of Zap70 pTyr^319^, Actin and Zap70 after antigen activation of T cells expressing each of the indicated receptors. Right: Heat map of mean band intensity of Zap70 pTyr^319^ normalized to actin loading control (n=3 independent experiments).

We designed two variations of CD19-specific ChTCRs, a “split” format in which the VH and VL fragments of the CD19-specific antibody (FMC63) were fused to TRAC and TRBC chains of the TCR respectively, and a novel “full” ChTCR format where the scFv (V_L_-linker-V_H_) was fused to TRAC while TRBC was co-expressed in the vector but left void of a ligand binding domain (Fig. 1a **and Extended Data Fig 1a**). For comparison, we utilized previously described CD19-specific CD28/CD3ζ and 4–1BB/CD3ζ CARs (Fig. 1a **and Extended Data Fig 1a**).

Split and full CD19 specific ChTCRs and CARs were expressed in CD8^+^ T cells by lentiviral gene delivery, followed by knockout of endogenous TCRαβ chain expression using cytosine base editors (CBE) to avoid mispairing of the ChTCR with endogenous TCR chains (**Extended Data Fig. 1b**). TCR^ko^ efficiency was greater than 90% for both TCR chains and improved the expression level and frequency of split and full ChTCR^+^ T cells compared to no knockout and single TRAC or TRBC KO (Fig. 1b–c **and Extended Data Fig. 1c-d)**. Expression of ChTCRαβ and CD3ε were restricted to split and full ChTCR^+^ T cells demonstrating association of ChTCRs with endogenous CD3ε (Fig. 1d–e). CD3ε expression on full ChTCR^+^ T cells was significantly higher than on split ChTCR^+^ T cells and comparable to unedited T cells, suggesting possible differences in the efficiency of assembly or trafficking between the two ChTCR formats (Fig. 1e). Confirmation that the ChTCRs associated with all endogenous CD3 subunits was shown by tagging ChTCR and CAR receptors with HA, before immunoprecipitation with anti HA and immunoblotting for individual CD3 subunits **(Extended Data Fig. 2)**.

T cells form a well-organized synapse with target cells expressing cognate peptide/MHC (p/MHC) that serves to amplify and regulate signaling [32, 33]. TCRs are concentrated in a central supramolecular complex (cSMAC) with adhesion molecules such as LFA-1, a ligand for ICAM-1, at the periphery of the synapse (pSMAC), and the CD45 phosphatase further excluded to the distal SMAC. We used TIRF microscopy to examine immune synapses formed between ChTCR and CAR expressing T cells and soluble lipid bilayers containing fluorescently labeled CD19 and ICAM-1. The synapses of CD28/ζ and 4–1BB/ζ CD19 CAR T cells were disorganized with CD19 and ICAM-1 intertwined across the synapse, as previously described [34]. In contrast split and full ChTCR T cells formed an organized cSMAC containing CD19 surrounded by a peripheral ring of ICAM-1 similar to that described for a TCR (Fig. 1f and 1g).

Intracellular signaling after antigen recognition by CARs is distinct from that mediated by TCR recognition of p/MHC [14, 15]. In the absence of antigen, many CARs exhibit tonic signaling, which can drive T cell exhaustion, limit T cell persistence *in vivo,* and increase toxicities [35, 36]. To evaluate tonic signaling of ChTCRs, we transduced Jurkat triple reporter cells with split and full CD19 ChTCRs, 28/ζ and 4–1BB/ζ CARs, and an NY-ESO-1 TCR. A low frequency of cells expressing the TCR, 4–1BBζ CAR or either of the ChTCRs showed NFAT and NFkB activation, whereas a high frequency of cells expressing the 28ζ CAR activated NFAT and NFkB in the absence of antigen recognition **(Extended Data Fig. 3a-c)**. We evaluated signaling in primary T cells expressing CD19-specific ChTCRs and CARs by first measuring calcium influx after crosslinking receptors with CD19 antigen. Split and full ChTCRs and the 28ζ CAR fluxed calcium with similar magnitudes, whereas calcium influx was barely detectable in T cells expressing the 4–1BBζ CAR (Fig 1h). We then compared phosphorylation of LAT and Zap70 using antigen coated beads to activate CAR, ChTCR, or TCR expressing T cells. Activation of T cells expressing the split and full ChTCRs led to the rapid phosphorylation of LAT Y220 and Y171 and Zap70 Y319 at the one minute time point, matching the kinetics of the NY-ESO 1 TCR (Fig 1i, j **and Extended Data Fig. 3d)**. In contrast, T cells expressing CARs showed less intense LAT and Zap70 phosphorylation that peaked later after 5 minutes. These data indicate that like a TCR, both ChTCR formats assemble with all CD3 chains, form TCR-like synapses, lack tonic signaling, and induce rapid antigen-specific signaling.

## The CD19 full ChTCR has superior antigen sensitivity and anti-tumor efficacy

**Figure 2.**
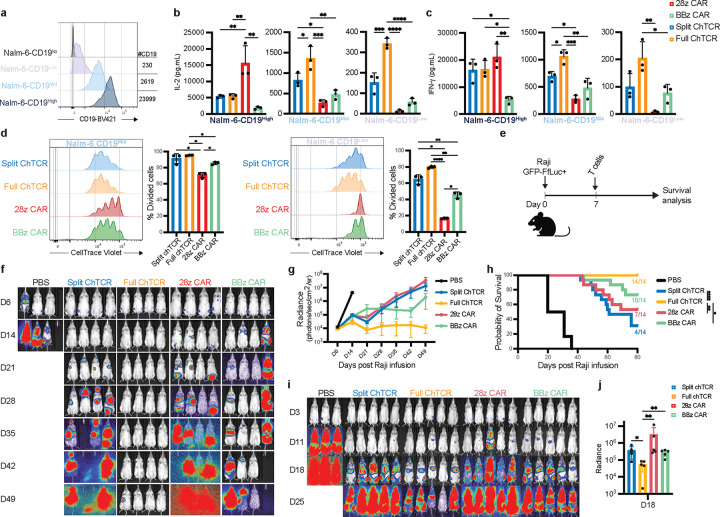
T cells expressing the CD19-specific Full ChTCR recognize CD19 low tumor cells and have superior anti-tumor effect in vivo **a.** Flow histograms of CD19 expression in Nalm-6 cells with varying levels of CD19. **b.** Concentration of IL-2 in culture supernatant after overnight co-culture of T cells expressing each of the indicated receptors with Nalm-6 CD19^High^, CD19^Mid^, and CD19^Low^ cells at an effector to target ratio (E:T) of 1:1. Data is shown as the mean ± SD for 3 independent experiments. *P<0.05, **P<0.01, ***P<0.001, ****P<0.0001 by two-way ANOVA **c.** Concentration of IFN-γ in culture supernatant for each experimental group described in **1b**. Data is shown as the mean +/SD for 3 independent experiments. *P<0.05, **P<0.01, ***P<0.001 by two-way ANOVA. **d.** Left: Representative histograms of CellTrace Violet (CTV) dye dilution in T cells expressing the indicated receptors measured after 3 days of co-culture with Nalm-6 CD19^Mid^ and Nalm-6 CD19^Low^ tumor cells. Right: Percent of divided cells (mean ± SD) for 3 independent experiments, *P<0.05, **P<0.01, ****P<0.0001 by two-way ANOVA. **e.** Schematic of the Raji GFP-ffluc^+^ NSG mouse model. **f.** Representative bioluminescence images of Raji-ffluc tumor burden in NSG mice treated with 2×10^6^ T cells expressing each of the indicated receptors. **g.** Tumor burden (mean ± SD radiance (photons/sec/cm^2^/steradian) of Raji GFP-ffluc^+^ bearing NSG mice treated as described in **f**. (n=4 or 5 mice per group; 3 independent experiments). **h.** Kaplan-Meier survival of Raji GFP-ffluc^+^-bearing NSG mice treated as described in **2.f** (n=14 mice per treatment group). *P <0.05, **P<0.01, ***P<0.001. **i.** Representative bioluminescence images of NSG mice inoculated with Nalm-6 CD19^low^ GFP-ffluc^+^ tumor cells and treated with T cells expressing the indicated receptors. **j.** Tumor burden (mean ± SD radiance (photons/sec/cm^2^/steradian)) of Nalm-6 CD19^low^ GFP-ffluc^+^-bearing NSG mice 18 days after treatment with T cells expressing the indicated receptors (n=5 mice per group). *P <0.05, **P<0.01 by two-tailed unpaired t-test.

To determine the antigen sensitivity of full and split ChTCR^+^ T cells, we generated Nalm-6 cells expressing high, medium, and low levels of CD19 and analyzed recognition by primary T cells transduced with CD19 specific ChTCRs and CARs (Fig 2a). When co-cultured with CD19^high^ cells, CD28ζ CAR T cells produced greater levels of IL-2 and IFNγ compared to 4–1BBζ CAR and ChTCR T cells (Fig 2b and 2c). However, when co-cultured with Nalm-6 cells expressing medium or low levels of CD19, split and full ChTCR T cells produced more IL-2 and IFN-γ than either CAR format. Strikingly, T cells expressing the full ChTCR produced higher levels of IL-2 and INF-γ than T cells expressing the split ChTCR (Fig 2b and 2c). There was no difference in proliferation of CAR and ChTCR T cells when co-cultured with CD19^high^ cells, however ChTCR T cells proliferated more than CAR T cells when co-cultured with CD19^mid^ and CD19^low^ Nalm-6 cells, and full ChTCR T cells proliferated more than split ChTCR T cells in response to the CD19^low^ tumor (Fig 2d). Thus, both ChTCR formats confer improved recognition of low antigen expressing tumor cells compared to CARs, and the full ChTCR was superior to the split ChTCR.

To determine the antitumor activity of full ChTCR^+^ T cells in vivo, we treated NSG mice engrafted with Raji lymphoma cells with a low dose of each of the transduced T cells (Fig 2e). T cells expressing the full ChTCR quickly eradicated tumor cells in all treated mice and improved survival compared to mice treated with split ChTCR, CD28ζ or 4–1BBζ CAR T cells (Fig 2f–h). We further tested ChTCR^+^ T cells for antigen sensitivity *in vivo* by engrafting NSG mice with Nalm-6^low^ cells. T cells expressing both ChTCRs exhibited superior antitumor activity early after infusion compared to T cells expressing conventional CD28ζ or 4–1BBζ CARs, and the full ChTCR was again superior to the split ChTCR (Fig 2i, j). These data show that ChTCRs are more sensitive for recognizing antigen low tumor cells compared to conventional CARs and that the full ChTCR is the most effective receptor in vitro and *in vivo*.

## Design of a sensitive CD22 monospecific ChTCR

The TRBC chain in the CD19 specific full ChTCR was left unoccupied to allow targeting of a second antigen by fusing a scFv of different specificity to TRBC. Bispecific targeting of CD19 and CD22 with T cells has been a major interest in the clinic because CD19 and CD22 negative or low relapses occur after monospecific therapies targeting these antigens [8, 37]. Unfortunately, a CD19/CD22 bispecific CAR, termed the “Loop” CAR, demonstrated compromised sensitivity to each antigen compared to the respective monospecific CARs, and patients treated with the Loop CAR relapsed with CD19^low/neg^ CD22^+^ tumor cells [8]. We sought to determine if the ChTCR platform could provide a more effective bispecific CD19/CD22 receptor.

**Figure 3.**
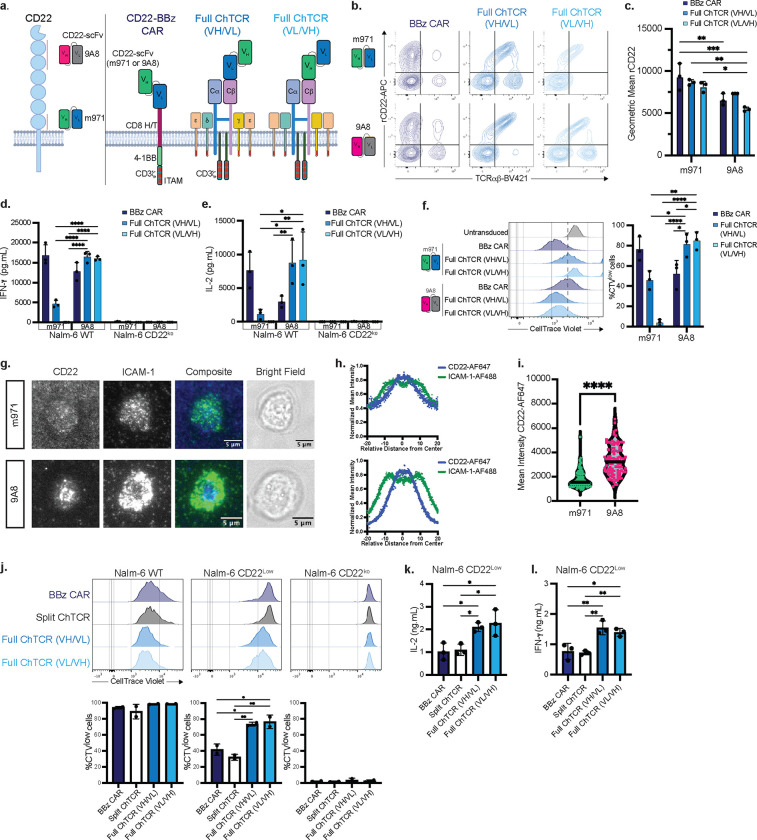
Design of a sensitive CD22-specific Full ChTCR. **a.** Left: Schematic of the CD22 molecule with locations of epitopes recognized by m971 and 9A8 scFvs. Right: Structure of CD22-specific receptors: CD22-BBz CAR: anti-CD22 scFV V_H_-V_L_ (m971 or 9A8), CD8 hinge and transmembrane domain, 4–1BB co-stimulatory domain, and CD3ζ domain; CD22 Full ChTCRs: m971 or 9A8 in VH/VL or VL/VH orientations linked to TRBC and shown associated with endogenous CD3 subunits. **b.** Representative flow plots of primary CD8 T cells stained with anti-TCRαβ and recombinant CD22 protein after lentiviral transduction and base editing to knock-out TCRαβ. Top row: T cells expressing CAR and ChTCRs constructed with the m971 scFV; Bottom row: T cells expressing CAR and ChTCRs constructed with the 9A8 scFv. **c.** Geometric mean ±SD of recombinant CD22-APC binding to CAR and ChTCR T cells constructed with either m971 or 9A8 scFv. Data is shown for T cells from 3 independent donors. *P< 0.05, **P< 0.01, ***P< 0.001 by two-way ANOVA. **d and e:** Concentration of IFN-γ (d) and IL-2 (e) in culture supernatant after overnight co-culture of T cells expressing each of the indicated receptors with Nalm-6^WT^ or CD22^ko^ cells. Data is shown as the mean ±SD for T cells from 3 independent healthy donors. *P< 0.05, **P< 0.01, ****P< 0.0001 by two-way ANOVA. **f.** Left: Representative histograms of CellTrace Violet (CTV) dilution measured after 72h co-cultures of T cells expressing each of the indicated receptors with Nalm-6 cells. Right: Frequency of divided cells. Data is shown as the mean ± SD for 3 independent healthy donors. *P< 0.05, **P< 0.01, ****P< 0.0001 by two-way ANOVA. **g.** Representative TIRF microscopy images of CD22 ChTCR T cells constructed with the m971 scFv (top) or 9A8 scFv (bottom) interacting with a soluble lipid bilayer functionalized with ICAM-1 extracellular domain (green) and CD22 extracellular domain (blue), or brightfield. Scale bars = 5μm. **h.** Normalized mean intensity of ICAM-1-AF488 and CD22-AF647 staining across cell radiuses in synapses; dots represent mean at each position, with trend line (n=100 cells). **i.** Mean intensity of CD22-AF647 staining within the synapse of 100 T cells expressing ChTCRs constructed with the m971 scFv or 9A8 scFv. **** P<0.0001 by two-tailed t-test. **j.** Top: Representative flow histograms of CellTrace Violet (CTV) dilution measured after 72h co-culture of T cells expressing the indicated receptors with Nalm-6 cells expressing different levels of CD22 or CD22 knockout. Bottom: Frequency of divided cells in each group. Data is shown as the mean ± SD for 3 independent healthy donors. *P< 0.05, **P< 0.01, ****P< 0.0001 by two-way ANOVA. **k and l.** Concentration of IL-2 (k) and IFN-γ (l) in culture supernatant after overnight co-culture with Nalm-6 CD22^low^ cells. Data is shown as the mean ± SD for 3 independent experiments. *P<0.05 and **P< 0.01 by 2-way ANOVA test.

We first evaluated monospecific full ChTCRs in which two different CD22 scFvs were fused to TRBC (Fig 3a). We tested the m971 scFv, which is used in the Loop CAR and targets a membrane proximal epitope, and the 9A8 scFv that targets a more membrane distal epitope (Fig 3a) [38]. ChTCRs were constructed in both V_H_V_L_ and V_L_V_H_ orientations, expressed in TRBC and TRAC edited T cells, and compared to conventional 4–1BBζ CARs constructed with the same scFvs (Fig 3a). CD8^+^ T cells expressing all constructs bound soluble recombinant CD22, with the m971 CAR and ChTCR showing a higher MFI of CD22 binding than observed with 9A8 (Fig 3b,c). However, when T cells were co-cultured with WT Nalm-6 cells, m971 ChTCR^+^ T cells produced lower levels of IL-2 and INF-γ, and proliferated poorly compared to T cells expressing the 9A8 ChTCR (Fig 3d–f; **Extended Data Fig. 4a)**. CAR T cells constructed with each scFv were functional in these assays, with m971 CAR T cells producing higher levels of cytokines and proliferating better than 9A8 CAR T cells (Fig 3d–f). A ChTCR in which the m971 scFv was fused in VL/VH or VH/VL orientations to the TRAC chain rather than TRBC also bound soluble CD22 but did not function against CD22^+^ tumor cells (**Extended Data Fig. 4b-f**). The discrepancy between binding of soluble CD22 by m971 and 9A8 ChTCR T cells and function in response to tumor cells suggested that the epitope targeted by m971 on membrane bound CD22 is less accessible. This was supported by analysis of synapses formed by CD22-specific ChTCR T cells with lipid bilayers functionalized with CD22 and ICAM-1. T cells expressing the 9A8 ChTCR formed a synapse with CD22 localizing to the center surrounded by a ring of ICAM-1 whereas T cells expressing the m971 ChTCR showed minimal CD22 accumulation in the synapse (Fig 3g–i).

We next compared antigen binding and sensitivity of T cells expressing the 9A8 full or split ChTCRs or a control 4–1BBζ CAR (**Extended Data Fig. 5a).** Binding to CD22 was similar for the full ChTCR and 4–1BBζ CAR T cells and higher than that observed with split ChTCR T cells (**Extended Data Fig. 5b, c).** T cells expressing the full ChTCR also showed higher TCRαβ expression than T cells expressing the split ChTCR (**Extended Data Fig. 5d**). To evaluate antigen sensitivity, we used Nalm-6 CD22^WT^ (11912 molecules/cell) and CD22^Low^ cell lines (959 molecules/cell) that were derived as target cells (**Extended Data Fig. 5e**). T cells expressing the full and split ChTCRs and the 4–1BBζ CAR proliferated similarly after co-culture with Nalm-6^WT^ cells (Fig 3j), however T cells expressing the full ChTCR demonstrated greater proliferation compared to split ChTCR^+^ and CAR^+^ T cells after co-culture with Nalm-6 CD22^Low^ cells. The full ChTCR T cells also produced higher levels of IL-2 and IFN-γ in response to Nalm-6 CD22 ^Low^ cells compared to split ChTCR and 4–1BBζ CAR T cells (Fig 3k, l). These findings showed that the CD22 full ChTCR provided more sensitive recognition of CD22 on tumor cells than the CD22 split ChTCR and 4–1BBζ CAR. This data also provided the rationale to investigate whether a ChTCR designed with CD19 and CD22 scFvs fused to each individual TCR chain could provide sensitive bispecific antigen recognition.

## Design and function of bispecific CD19/CD22 ChTCR

**Figure 4.**
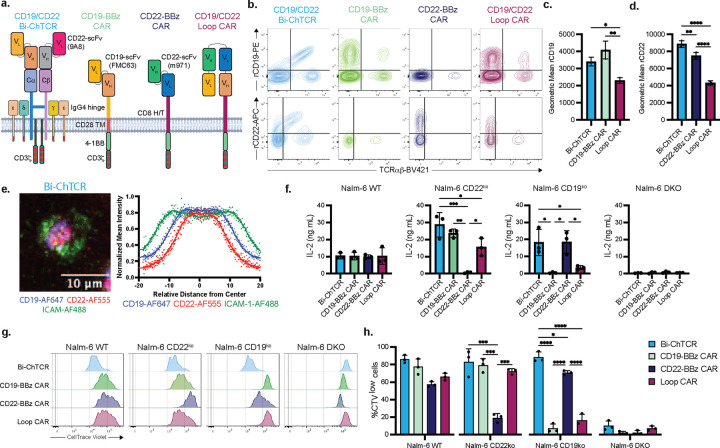
A CD19/CD22 Bi-ChTCR confers T cell recognition of both CD19 and CD22. **a.** Schematic of bispecific CD19/CD22 ChTCR, monospecific CD19 and CD22 4–1BB/CD3ζ CARs, and bispecific CD19/CD22 CAR [7]. The CD19-specific FMC63 scFv and the CD22-specific 9A8 scFv were used in the Bi-ChTCR construct; FMC63 and m971 scFvs were used in all CAR constructs. **b.** Representative flow plots of transduced and TCRαβ base edited primary CD8 T cells stained with anti-TCRαβ and recombinant CD19 (top) or CD22 proteins (bottom). **c. and d.** Geometric mean ± SD of CD19-PE (c) or CD22-APC (d) binding to T cells expressing the indicated receptors (n=3 independent experiments). *P< 0.05, **P< 0.01, ****P< 0.0001 by two-way ANOVA. **e.** Left: Representative TIRF microscopy image of CD19/CD22 Bi-ChTCR T cells interacting with a soluble lipid bilayer functionalized with ICAM-1 extracellular domain (green), CD19 and CD22 extracellular domains (blue and red respectively). Scale bars = 10μm. Right: Normalized mean intensity of ICAM-1-AF488, CD19-AF647 and CD22-AF555 staining across the cell radiuses in synapses. Dots represent mean at each position, with trend line (n=100 cells). **f.** Concentration of IL-2 in culture supernatants after overnight co-culture of T cells expressing the indicated receptors with Nalm-6^WT^, Nalm-6^CD22ko^, Nalm-6^CD19ko^ and Nalm-6^DKO^ cells. Data is shown as the mean ± SD for T cells from 3 independent healthy donors. *P< 0.05, **P< 0.01, ***P< 0.001 by two-way ANOVA. **g.** Representative flow histograms of CellTrace Violet (CTV) dilution measured after 72h co-culture of T cells expressing the indicated receptors with Nalm-6 cells expressing CD19 and CD22 or knocked out for one or both targets. **h.** Frequency of divided cells (CTV^low^ cells) measured after 72h co-culture of T cells expressing the indicated receptors with Nalm-6 cells. Data is shown as the mean ± SD from 3 independent healthy donors. *P< 0.05, ***P< 0.001, ****P< 0.0001 by two-way ANOVA.

We constructed a lentiviral vector that encoded the CD19-specific FMC63 scFv fused to TRAC in a V_H_V_L_ orientation (Fig 1a) and the CD22 specific 9A8 scFv fused to TRBC in a V_L_V_H_ orientation (Fig 4a; **Extended Data Fig. 6a).** CD8^+^ T cells were transduced with this Bi-ChTCR followed by TCR KO using CBE. For functional comparison, T cells were transduced with CD19 monospecific 4–1BBζ CAR, CD22 monospecific 4–1BBζ CAR using the m971 scFv, and with the bispecific Loop CAR [37, 38] (Fig 4a–b). The MFI of rCD19 and rCD22 binding was superior for T cells expressing the Bi-ChTCR compared to the Loop CAR (Fig 4b–d).

Expressing two scFvs on the same ChTCR could result in interactions between the scFvs that induce tonic signaling or affect synapse formation. When expressed in Jurkat TPR cells, Bi-ChTCR exhibited minimal antigen-independent activation as demonstrated by the frequency of NFAT^+^, NF-kB^+^ and AP-1^+^ cells **(Extended Data Fig. 6b-d)**. Monospecific CD19 and CD22 4–1BBζ CARs also had minimal tonic signaling in Jurkat TPR cells while the Loop CAR exhibited a high level of antigen-independent activation **(Extended Data Fig. 6b-d)**. Additionally, the Bi-ChTCR formed an organized synapse when tested on a soluble bilayer functionalized with CD19 and CD22 proteins with CD22 taking a more central position than CD19 in the cSMAC (Fig 4e).

We next transduced primary T cells and tested their recognition of Nalm-6 cells that expressed endogenous levels of CD19 and CD22, Nalm-6 cells that were gene edited to express only CD19 (Nalm-6 CD22^ko^), only CD22 (Nalm-6 CD19^ko^), or neither CD19 and CD22 (Nalm-6 DKO) (**Extended Data Fig. 6e**). Bi-ChTCR T cells demonstrated robust proliferation and cytokine production in response to Nalm-6^WT^ and Nalm-6 cells expressing only CD19 or CD22 (Fig. 4f–h **and Extended Data Fig. 6f**). In contrast, Loop CAR T cells showed reduced functions when co-cultured with CD19^ko^ cells compared to Nalm6 WT or CD22^ko^, and CD19 and CD22 mono-specific CAR T cells only proliferated and produced cytokines when co-cultured with cells expressing their cognate antigen (Fig. 4f–h **and Extended Data Fig. 6f)**. T cells expressing both Bi-ChTCRs lysed Nalm-6 WT, Nalm-6 CD22^ko^, and Nalm-6 CD19^ko^ cells, while T cells expressing the Loop CAR lysed Nalm-6 CD22^ko^ cells but exhibited poor lysis of Nalm-6 CD19^ko^ target cells (**Extended Data Fig. 6g)**. As expected, monospecific CAR T cells failed to recognize Nalm-6 cells lacking the cognate antigen (**Extended Data Fig. 6g**).

**Figure 5.**
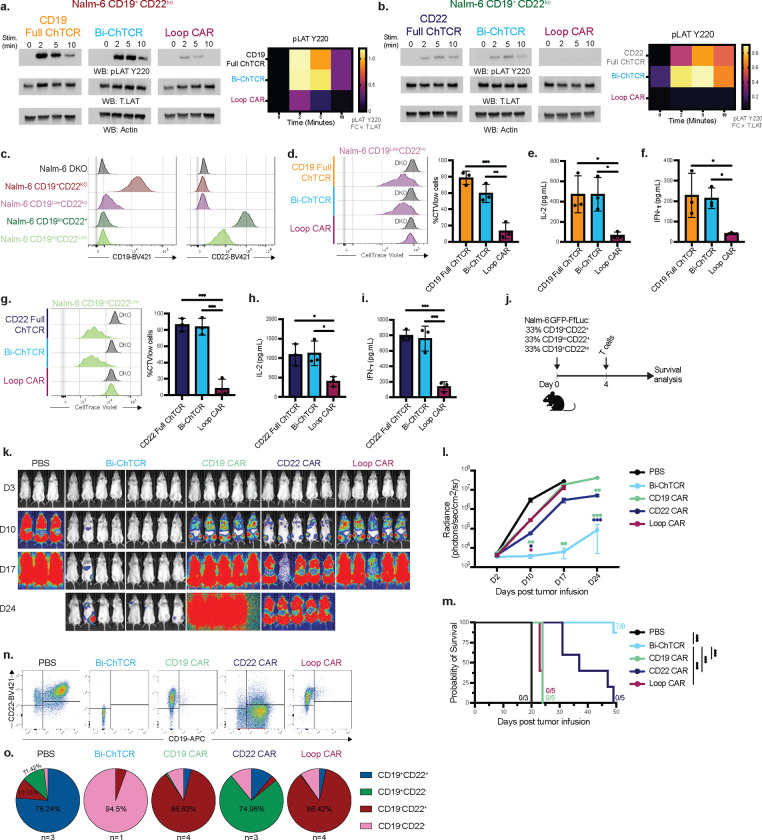
T cells expressing the CD19/CD22 Bi-ChTCR have exquisite sensitivity for both antigens and potent anti-tumor activity. **a. and b.** Representative western blot for LAT pTyr^220^, Actin and LAT in lysates from T cells expressing the indicated receptors after stimulation with Nalm-6 CD19^+^CD22^ko^ cells (a) or Nalm-6 CD19^ko^CD22^+^ cells (b) for the indicated times. Right: Heat map of mean band intensity of LAT pTyr^220^ normalized to actin loading control (n=3 independent experiments). **c.** Flow plots depicting CD19 and CD22 expression levels on Nalm-6 cells after gene knockout. **d.** Left: Representative flow histograms of CellTrace Violet (CTV) dilution measured after 72h co-culture with Nalm-6 cell CD19^ko^CD22^ko^ (DKO, control) or Nalm-6 CD19^Low^CD22^ko^ cells. Right: Frequency of divided cells (CTV^low^ cells) measured after 72h co-culture with Nalm-6 CD19^Low^CD22^ko^ cells (mean ± SD) (n=3 independent healthy donors). P values from two-way ANOVA test, **P< 0.01, ***P< 0.001. **e-f.** Concentration of IL-2 (e.) and IFN-γ (f.) in culture supernatant after overnight co-culture with Nalm-6 CD19^Low^CD22^ko^ cells (mean ± SD) (n=3 independent healthy donors). P values from two-way ANOVA statistical test, *P< 0.05. **g.** Left: Representative flow histograms of CellTrace Violet (CTV) dilution measured after 72h co-culture with Nalm-6 DKO cells or Nalm-6 CD19^ko^CD22^Low^ cells. Right: Frequency of divided cells (CTV^low^ cells) measured after 72h co-culture with Nalm-6 CD19^ko^CD22^Low^ cells (mean ± SD) (n=3 independent healthy donors). P values from two-way ANOVA statistical test, **P< 0.01, ***P< 0.001. **h-i.** Concentration of IL-2 (h) and IFN-γ (i) in culture supernatant after overnight co-culture with Nalm-6 CD19^ko^CD22^Low^ cells (mean ± SD) (n=3 independent healthy donors). P values from two-way ANOVA test, *P< 0.05. **j.** Schematic of NSG mice engrafted with Nalm-6 GFP-ffluc^+^ cells that are heterogeneous for CD19 and CD22 expression. **k.** Representative bioluminescence images of Nalm-6 GFP-ffluc^+^ tumor burden in mice treated with 2×10^6^ T cells expressing the indicated CARs or the Bi-ChTCR. **l.** Tumor burden (mean ± SD radiance (photons/sec/cm^2^/steradian)) of tumor-bearing NSG mice treated with T cells expressing the indicated CARs or the Bi-ChTCR (n=5 to 8 mice per group). *P <0.05, **P<0.01, ***P< 0.001 by two-way ANOVA. **m.** Kaplan-Meier survival of Nalm-6 GFP-ffluc^+^-bearing NSG mice treated with CARs or Bi-ChTCRs (n=5–8). *P <0.05, **P<0.01, ***P<0.001. **n. and o.** Representative flow plots (n) and pie graphs (o) of CD19 and CD22 expression on Nalm-6 GFP-ffluc+ tumor cells harvested from the bone marrow at the time of euthanasia for each of the treatment groups (n=3–4 mice per group).

It was conceivable that signaling or sensitivity of the Bi-ChTCR for each single antigen would be compromised by the presence of two different scFvs in close proximity. We measured LAT phosphorylation after co-culturing T cells expressing individual monospecific and Bi ChTCRs, or the Loop CAR with CD19 or CD22 positive Nalm-6 cells. LAT phosphorylation was comparable in intensity and kinetics between the Bi-ChTCR and the monospecific CD19 or CD22 full ChTCRs, and greater than that observed with the Loop CAR (Fig. 5a–b). To evaluate antigen sensitivity, we compared T cell recognition of Nalm-6 CD22^ko^ cells expressing high and low levels of CD19, and of Nalm-6 CD19^ko^ cells expressing high and low levels of CD22 (Fig 5c). Strikingly, T cells expressing the CD19/CD22 Bi-ChTCR exhibited strong proliferation (Fig 5d and g) and cytokine production against both CD22^ko^CD19^low^ and CD22^low^CD19^ko^ tumor cells (Fig 5e, f, h and i) that was equivalent to mono-specific CD19 and CD22 full ChTCRs and superior to the Loop CAR.

We next modeled in vivo therapy of NSG mice engrafted with a mixture of Nalm-6^WT^ (CD19^+^/CD22^+^), Nalm-6 CD19^ko^/CD22^+^, and Nalm-6 CD19^+^/CD22^ko^ tumor cells (Fig. 5j). Mice treated with bi-ChTCR T cells showed improved tumor clearance and survival compared to mice treated with monospecific or bispecific CAR T cells (Fig. 5k–m). We harvested tumor cells at euthanasia due to tumor progression to determine the expression of CD19 and CD22 on tumor cells (Fig. 5n–o). In control untreated mice, tumor cells were predominantly CD19^+^CD22^+^ with a smaller frequency of CD19^+^ CD22^−^ and CD19^−^ CD22^+^ cells than in the initial tumor inoculum, illustrating a proliferative advantage for Nalm-6^WT^ tumor cells in vivo. Mice that received CD19 CAR T cells or Loop CAR T cells relapsed with predominantly CD19^−^CD22^+^ tumor cells and minor populations of CD19^low^CD22^+^ and CD19^−^CD22^−^ tumor cells. In mice that received CD22 CAR T cells, the persisting tumor cells were predominantly CD19^+^CD22^−^ with a small frequency of CD19^+^ CD22^+/low^ and CD19^−^ CD22^−^ cells. Only one mouse progressed and was euthanized from the group that received Bi-ChTCR T cells and in this case the tumor cells were predominantly CD19^−^CD22^−^ with a small fraction of CD19^−^ CD22^Low^ cells. These data show that heterogeneity in antigen expression and antigen density limit antitumor activity after mono- and bispecific CAR T cell therapy, and these barriers to efficacy are overcome by sensitive Bi-ChTCR T cells.

## Bi-ChTCR specific for multiple myeloma antigens

To determine whether the Bi-ChTCR architecture could be used to target multiple myeloma, we designed BCMA/SLAMF7 Bi-ChTCRs. Multiple permutations were tested including fusing a BCMA-targeting scFv to TRAC and a SLAMF7-specific scFv to TRBC, both in VH/VL orientations (format #1), switching the pairing of scFv and TCR chains (format 2), and placing either the two scFvs in VL/VH orientation on each TCR chain (format 3) or the BCMA scFv in VH/VL and SLAMF7 scFv in VL/VH (format 4) (**Extended Data Fig. 7a**). All 4 Bi-ChTCRs were expressed in TCR^ko^ Jurkat cells at similar levels as measured by binding to rBCMA and SLAMF7, and assembled with CD3 as demonstrated by restored cell surface expression of CD3ε (**Extended Data Fig. 7b-e)**. These results illustrate the resilience of the ChTCR architecture for bispecific targeting of tumor antigens.

**Figure 6.**
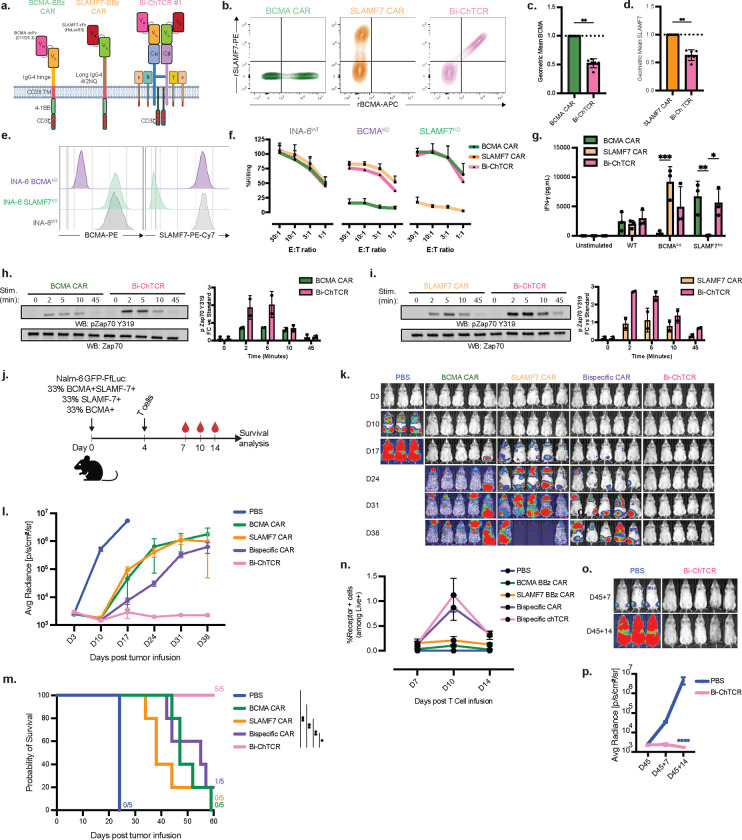
BCMA/SLAMF7 Bi-ChTCR for targeting Multiple Myeloma **a.** Schematic of CARs and a Bi-ChTCR specific for BCMA and SLAMF-7. The BCMA BBz CAR was constructed with the C11D5.3 scFV V_H-_V_L_ linked to an I_g_G4 hinge, CD28 transmembrane domain, and 4–1BB and CD3ζ signaling domains. The SLAMF7 BBz CAR was constructed with the HuLuc63 V_H_-V_L_ linked to a long IgG4 4/2NQ hinge, CD28 transmembrane domain, and 4–1BB and CD3ζ signaling domains; The BCMA/SLAMF7 Bi-ChTCR was constructed with the C11D5.3 scFV V_H-_V_L_ fused to TRAC and the HuLuc63 V_H_-V_L_ fused to TRBC. **b.** Representative flow plots of transduced and TCRαβ and SLAMF7 based edited primary CD8 T cells stained with recombinant SLAMF7-PE and BCMA-APC. **c. and d.** Normalized geometric mean ± SD of binding of BCMA-APC (c.) or SLAMF7-PE (d.) to CD8 T cells expressing each CAR or the Bi-ChTCR. Data is shown for 6 independent experiments. **P< 0.01 by paired t-test. **e.** Flow histograms of BCMA (left) and SLAMF7 (right) expression in INA-6 WT, INA-6 SLAMF7^KO^ and INA-6 BCMA^KO^ myeloma cells. **f.** Cytotoxic activity of CAR or Bi-ChTCR T cells against the indicated INA-6 cells measured by chromium release assay. Data is shown as the mean ± SD for 3 independent experiments. **g.** Concentration of IFN-γ in culture supernatant after overnight co-culture of T cells expressing the indicated receptors with wild-type and single antigen knock-out INA-6 cells. Data is shown as the mean ± SD for 3 independent experiments. P< 0.05, **P< 0.01, ***P< 0.001 by two-way ANOVA. **h and i.** Left: Representative western blot analysis of lysates from BCMA CAR and Bi-ChTCR (h) or SLAMF7 CAR and Bi-ChTCR (i) for pZap70 pTyr^319^ and Zap70 after crosslinking the receptor with antigen-coated beads. Right: Fold-change in mean intensity of pZap70 pTyr^319^ normalized to actin per stimulation timepoints. **j.** Schematic of NSG mice engrafted with Nalm-6 cells that are heterogeneous for BCMA and SLAMF7 antigen expression and treated with 2×10^6^ CAR or ChTCR T cells. **k.** Bioluminescence imaging of Nalm6-ffluc tumor burden in mice after treatment with T cells expressing monospecific or bispecific CARs or the Bi-ChTCR. **l.** Mean ± SD radiance (photons/sec/cm^2^/steradian) of Nalm-6 GFP-ffluc tumor burden for each treatment group (n=3–5 mice per group). **m.** Kaplan-Meier survival of Nalm-6 GFP-ffLuc-bearing mice in each treatment group (n=3–5 mice per group). *P<0.05, ** P <0.01. **n.** Frequency of CAR+ and Bi-ChTCR+ T cells in the blood at indicated timepoints (% of Live+Lymphocyte+). **o.** Bioluminescence images of tumor burden in mice rechallenged with a mixture of BCMA+SLAMF7+, BCMA-SLAMF7+ and BCMA+SLAMF7- Nalm-6 GFP-ffluc cells 45 days after initial tumor infusion. **p.** Mean of Nalm-6 GFP-ffluc tumor burden in new control group or mice previously treated with Bi-ChTCR T cells and rechallenged (mean radiance ± SD) (photons/sec/cm^2^/steradian) (n=3–5 mice per group). ****P<0.0001 by two-way ANOVA.

We proceeded with a detailed analysis of format 1 since the VH/VL orientation provided a direct comparison to previously designed BCMA and SLAMF7 CARs (**Extended Data Fig. 8a, Fig. 6a**). The BCMA/SLAMF7 Bi-ChTCR and monospecific BCMA and SLAMF7 CARs were expressed in primary CD8+ T cells with simultaneous CBE of endogenous TRAC and TRBC and SLAMF7 to eliminate endogenous TCR expression and to avoid fratricide since SLAMF7 is expressed on some T cells [39] (**Extended Data Fig. 8b-e**). Gene edited SLAMF7 and BCMA/SLAMF7 Bi-ChTCR T cells expanded in culture and had the same viability as BCMA CAR T cells. Binding of soluble BCMA and SLAMF7 was significantly higher for T cells transduced with the monospecific CARs compared to the Bi-ChTCR (Fig 6b–d). Functional studies showed that BCMA/SLAMF7 Bi-ChTCR T cells recognized the MM cell line INA-6 expressing both BCMA and SLAMF7, and INA-6 engineered to express only a single antigen, whereas monospecific CAR T cells only recognized INA-6 cells that expressed their cognate antigen (Fig. 6e–g). Bi-ChTCR T cells also eliminated a mixture of heterogenous Nalm-6 target cells that were BCMA^+^SLAMF7^ko^ and BCMA^ko^SLAMF7^+^ in vitro, unlike monospecific CAR T cells **(Extended Data Fig. 8g)**. Bi-ChTCR T cells exhibited more rapid and intense phosphorylation of Zap70 and LAT compared to monospecific BCMA and SLAMF7 CAR T cells (Fig. 6h and i). Importantly, despite the lower level of receptor expression, BCMA/SLAMF7 Bi-ChTCRs exhibited superior sensitivity for each antigen compared to monospecific CAR T cells, as demonstrated by higher levels of IFN-γ secretion when cultured with a range of concentrations of plate-bound antigen (**Extended Data Fig. 8h-i)**.

Before analyzing the in vivo function of T cells expressing the BCMA/SLAMF7 Bi-ChTCR, we compared expression and signaling to a previously described BCMA/SLAMF7 bispecific CAR in primary T cells [40]. We observed superior binding of BCMA and SLAMF7 to the Bi-ChTCR compared to the BCMA/SLAMF7 CAR (**Extended Data Fig. 9a-c**), and T cells expressing Bi-ChTCR exhibited more rapid and intense Zap70 phosphorylation than bispecific CAR T cells after stimulation with bead-coated BCMA and SLAMF7 alone, or together (**Extended Data Fig. 9d,e).** We then asked whether BCMA/SLAMF7 Bi-ChTCR^+^ T cells could eliminate a tumor inoculum comprised of Nalm-6 cells that were heterogeneous for BCMA and SLAMF7 expression in NSG mice (Fig. 6j). Bi-ChTCR^+^ T cells rapidly eliminated tumor in all mice, while the bispecific CAR^+^ T cells showed a moderate improvement in tumor control and survival over each of the mono-specific CAR^+^ T cell products (Fig. 6k–m). Bi-ChTCR T cells also demonstrated superior expansion in the blood during the period of tumor eradication compared to all other treatment groups, despite the absence of a costimulatory domain the receptor construct (Fig. 6n). We rechallenged mice that were tumor free after the administration of Bi-ChTCR with the same mixture of Nalm-6 cells. Control mice quickly developed tumors, whereas all mice from the Bi-ChTCR treated group were protected from tumor challenge (Fig. 6o–p). Collectively, the data demonstrates that Bi-ChTCRs can be designed to sensitively target pairs of B cell lineage antigens relevant to therapy of leukemia, lymphoma and multiple myeloma.

## Discussion

The outgrowth of tumor cells that have downregulated or lost the target antigen is a major mechanism for the failure of CAR T cell therapies [6, 8, 11, 18, 41, 42]. Improving efficacy requires the development of synthetic receptors that are highly sensitive and capable of recognizing multiple tumor antigens. Bispecific CARs have been designed for this purpose but often exhibit reduced sensitivity for each individual antigen, enabling escape of tumor cells with low antigen levels [8, 43]. Here, we describe the design and function of chimeric TCRs that simultaneously target two tumor antigens with high sensitivity.

The concept of linking an antigen binding domain to the TCR predates the design of current CARs, but was ineffective due to mispairing of ChTCR chains with endogenous TCR chains, which compromised the cell surface expression of the ChTCRs [31, 44, 45]. We employed base editing to reproducibly and efficiently disrupt endogenous TRAC and TRBC expression. Base editing may be a safer alternative to conventional CRISPR for multiplexed gene editing as it does not induce double-strand DNA breaks that can lead to chromosome losses, translocations and/or recombination events [46, 47]. Base editing can also be used to disrupt expression of multiple genes in T cells without loss of editing efficiency, as shown by the simultaneous knock-out of TRAC, TRBC, and SLAMF7 during generation of Bi-ChTCR T cells. The absence of endogenous TRAC and TRBC chains is required for optimal ChTCR expression to facilitate the assembly of all CD3 subunits with the ChTCR and provide diversity in ITAM sequences, which is crucial for optimal T cell activation [20, 48]. Recent studies have incorporated signaling portions from CD3ε, δ or γ subunits into CARs to manipulate CAR T cell function. CD3ε, in particular, tuned down T cell cytokine production, extended T cell persistence and prevented dysfunction in vivo [20, 49, 50]. However, the antigen sensitivity of these CD3ε modified CARs was not evaluated and tuning of T cell activation might reduce antigen sensitivity. The importance of recapitulating the TCR structure is illustrated by our synapse studies, which revealed that ChTCRs, unlike CARs, form a TCR-like bull’s eye synapse with ligand-functionalized lipid bilayers [51]. Immune synapses play a pivotal role in regulating T cell activation, from signal initiation, propagation and termination [52]. This regulation is likely to be preserved in ChTCR T cells that assemble with all CD3 chains and may be important in achieving optimal antitumor responses after ACT.

The full ChTCR format, in which an scFv is linked to a single TCR constant chain, demonstrated superior antigen sensitivity in vitro and antitumor activity in vivo compared to the split ChTCR format [30, 31]. We leveraged the superior monospecific full ChTCR format to design bispecific ChTCRs that target CD19 and CD22 or BCMA and SLAMF7. Given the absence of rules for designing optimized ChTCRs, we constructed monospecific CD22 ChTCRs with two CD22 scFvs that had similar binding affinities (m971 and 9A8) but were specific for membrane-proximal and membrane-distal epitopes, respectively [38, 53]. Surprisingly, while T cells expressing ChTCRs using each scFv bound soluble CD22 protein, only T cells expressing the 9A8 ChTCR formed an organized immune synapse and recognized CD22 positive tumor cells. In contrast, the m971 scFv specific for a membrane proximal epitope in CD22 was superior for CAR T cells [54]. This further highlights differences between CARs and ChTCRs that may be related to distinct synaptic distance requirements between the two receptor classes [55, 56]. Unlike CARs, ChCTRs do not possess a hinge domain between the TCR constant chains and the scFv, which could limit the flexibility necessary to engage the membrane-proximal epitope of a bulky protein like CD22. These findings underscore the need to define rules for optimal ChTCR design, informed by functional screening of scFv binders of known epitope specificity to facilitate structural analysis.

T cells expressing Bi-ChTCRs targeting CD19 and CD22 or BCMA and SLAMF7 recognized target cells expressing either one or both target proteins and exhibited superior sensitivity for tumor cells with low antigen levels compared to T cells expressing monospecific and bispecific CARs. In vivo models that mimic therapy of tumors with heterogeneous antigen expression showed improved efficacy of Bi-ChTCR T cells compared to bispecific CAR T cells targeting the same antigens [37, 40]. Bispecific targeting with CARs can also be achieved with a bicistronic vector or by the infusion of two mono-specific CAR products either at the same time or sequentially. These strategies weren’t formally compared here but would not be predicted to provide better antigen sensitivity, and in the case of two products, add manufacturing complexity and cost [43, 57]. Surprisingly the superior in vivo efficacy of the Bi-ChTCR T cells was observed in the absence of providing additional costimulation either in trans or intrinsic to the ChTCR [30, 31, 58]. Whether providing costimulation to Bi-ChTCR T cells would further improve efficacy remains to be evaluated. It is notable that mono and Bi-ChTCR^+^ T cells secrete lower levels of cytokines compared to conventional CD28ζ CAR T cells in response to high antigen expressing tumor cells. High cytokine levels correlate with the severity of cytokine release syndrome and neurotoxicities observed with CAR T cell therapy [59]. It is therefore reasonable to expect a better toxicity profile with ChTCRs. ChTCR^+^ T cells produce higher levels of cytokines and proliferate better against low antigen tumor cells than CAR T cells, enabling the antitumor response to be sustained and eradicate antigen low tumor cells.

Collectively, these data identify a new approach for sensitive and potent recognition of two target antigens with a single engineered T cell product that holds promise for reducing antigen escape and relapse in B cell malignancies and multiple myeloma. This strategy may also be applicable to solid tumors where tumor heterogeneity is prevalent and antigen levels may be lower than in B-cell malignancies. However, unlike B cell lineage antigens, many targets in solid tumors are also expressed on normal epithelial tissues, and receptors that are too sensitive may cause on target off tumor toxicity. Careful selection of target antigens with well-defined tumor-restricted expression profile will be essential to apply Bi-ChTCRs in solid tumors.

## Methods

### Cell Lines

Lenti-X 293T cell line was acquired from Takara Bio USA_._ Jurkat 76 TPR cells were previously described [1] and a kind gift from Dr. Mirjam Heemskerk (University Medical Center, Utrech). Nalm-6 (CRL-3273), Raji (CCL-86), INA-6 and Jurkat E6.1 (TIB-52) were acquired from American Type Culture Collection. Lenti-X 293T were maintained in complete culture media (DMEM (Gibco, 11965–092), 10% fetal bovine serum (Corning, 35–011-CV), 2mM L-glutamine (Gibco, 25030–081), 1x penicillin/streptomycin (Gibco, 15140–122), 25mM HEPES (Gibco, 15630080)). NALM-6, Raji, INA-6 and Jurkat cell lines were maintained in RPMI 1040 medium (Gibco, 22400–089) supplemented with 10% fetal bovine serum, 2mM L-glutamine, 1x penicillin/streptomycin. Cells were split every 2–3 days and replated at a density of 0.3–0.6×10^6^ cells/mL. All cell lines were routinely checked to ensure they were negative for mycoplasma contamination.

### Generation of Nalm-6 lines

Nalm-6 cell lines expressing various amount of CD19 and CD22 were generated as described previously [2]. Briefly, CD19, CD22, or both were knocked out of Nalm-6 cells using CRISPR Cas9, and negative cells were transduced with a lentiviral vector encoding either a truncated CD19 protein (extracellular and transmembrane domains only, Uniprot P15391). Briefly, 1×10^6^ cells were resuspended in SF buffer (Lonza, V4XC-1032) mixed with 20nM of ribonucleoprotein protein complex formed with CRISPR Cas9 enzyme (Horizon Discovery, CAS12207) and sgRNAs of interest (Horizon Discovery). NALM-6 cells negative for the antigen of interest were sorted by flow cytometry, transduced with the lentiviral vector, subjected to single cell flow sorting to obtain different levels of CD19 and CD22 expression, and expanded for anlaysis. Nalm-6 cells expressing BCMA and/or SLAMF7 were generated by transduction with a lentiviral plasmid encoding for the extracellular and transmembrane domains of BCMA (Uniprot: Q02223) or SLAMF7 (Uniprot: Q9NQ25) and sorted by flow cytometry for purity.

### Generation of constructs and Lentivirus preparation

Lentivirus vector (HIV7) was used for transduction of T cells. CAR and ChTCR sequences were synthesized after codon optimization and inserted into the HIV7 lentivirus plasmid backbone under the EF1α promoter by Gibson assembly, or in some cases full plasmids were synthesized commercially (Twist Biosciences). Sequences for scFvs were previously described; anti-CD19 FMC63 [3], anti-CD22 m971 [4], anti-CD22 9A8 [5], anti-BCMA C11D5.3 [6], and anti-SLAMF7 HuLuc63 [7]. To generate split ChTCRs, human TCR constant beta chain (UniProt P01850, amino acids 1–176) was inserted immediately after the antibody V_L_ chain. A furin site, a P2A sequence and a GM-CSF signal peptide were inserted between the TCR chains. The TCR constant alpha chain sequence (UniProt P01848, amino acids 1– 140) was inserted immediately after the antibody V_H_ sequence from the scFv. Full ChTCR were designed by expressing the TRBC chain, fused to a furin site, a P2A sequence and a GM-CSF signal peptide before the entire scFv sequence (V_L_-linker-V_H_) and the TRAC chain. For Bispecific ChTCRs, each target-specific scFv was fused to a TCR chain. TRBC S56C and TRAC T47C substitutions were made to improve chain pairing [8]. In some experiments, the CAR and ChTCR constructs included an HA tag to facilitate immunoprecipitation. Replication-deficient lentivirus was produced by transient transfection of Lenti-X cells using pPAX2, pVSVG and receptor-encoding lentiviral vector using Xfect polymer transfection reagent (Takara Bio, 631318), according to the manufacture’s protocol. Lentiviral supernatant was harvested after 48 hours and filtered using a 0.45-mm PES syringe filter. Virus was further concentrated with Lenti-X Concentrator (Takara Bio, 631232) according to the manufacturer’s recommended protocol.

### T cell isolation

Peripheral blood was collected from healthy adults enrolled in IRB approved study at Fred Hutchinson Cancer Center or obtained from Bloodworks Northwest after informed consent. Peripheral blood mononuclear cells (PBMCs) were isolated by density gradient using SepMate-50 (Stem Cell Tech., 85450) and lymphocyte separation media (Corning, 25–072-CV). Bulk CD8+ and CD4+ T cells were isolated using EasySep T cell Isolation kit (Stem Cell Tech, 17953) following manufacturer’s instructions. T cells were cryopreserved for later use.

### T cell transduction and gene editing

Bulk CD8^+^ and CD4^+^ T cells were activated using Dynabeads Human T-Activator CD3/CD28 (Gibco, 11131D) at a 3:1 bead to T cell ratio. T cells were cultured in T cell media (CTL) (RPMI 1040 (Gibco, 22400–089), 10% Human serum (Bloodworks Northwest) 2mM L-glutamine (Gibco, 25030–081), 1x penicillin/streptomycin (Gibco, 15140–122), 0.5mM β-mercaptoethanol) supplemented with IL-2 (50 IU/mL). The next day concentrated lentiviral supernatant was added to activated T cells with LentiBOOST Solution B (100x) (SIRION Biotech SB-P-LV-101–12) and polybrene (Millipore, TR-1003-G) at a final concentration 4.4μg/mL. T cells were spinoculated at 800g, 32° C for 90 min, after overnight incubation beads removed prior to gene editing. Cytidine base editing was performed to knock-out expression of endogenous TRAC, and TRBC, both, and SLAMF7 in some experiments. 1×10^6^ T cells were resuspended in P3 buffer (Lonza, V4XP-3032), mixed with 1μg concentration of RNA guide and 1.5μg of CBE BE4max mRNA (Addgene plasmid 112093) (Aldevron), and electroporated using the Lonza 4D device (Lonza) [9]. Sequences for sgRNAs are described in Table S1. T cells were cultured in CTL supplemented with IL-2 (150IU/mL), IL-7 (5ng/ml), IL-15 (5ng/mL) initially, and then maintained in CTL supplemented with IL-2 (50IU/mL) for one week before being used for assays. For assays requiring larger cell numbers (western blot analysis and Calcium flux assays), flow sorted CAR and ChTCR specific T cells were expanded using OKT3 (30ng/mL) or PHA-L (500x) (Thermo Fisher Scientific, 00–4977-93), γ-irradiated lymphoblastoid cell line (LCL) (8000 rad), and γ-irradiated PBMCs at a LCL to T cell ratio (100:1) and PBMC to T cell ratio (600:1). IL-2 was added 24-hours after co-culture, and OKT3 or PHA-L was washed out on day 4. Cultures were fed with CTL supplemented with IL-2 (50IU/mL) and rested without IL-2 addition before use in assays.

### Flow Cytometry

T cells were stained to detect CAR or ChTCR expression with the appropriate recombinant proteins (Acro bioystems, CD19 (CD9-H82E9), BCMA (BCA-H82E4), CD22 (Siglec-2) (SI2-H82E3) and SLAMF7 (HL7-H82E0)), and with anti-TCRα/β-BV421 1:50 (BD, 744778) and anti-CD3ε-BUV395 1:50 (BD, 563546) antibodies. When proteins directly conjugated to a fluorescent label was not available, biotinylated recombinant protein was used, followed by incubation with fluorescently labeled streptavidin (BioLegend, APC (405207), PE (405204), BV421 (405226), BD Biosciences BUV395 (564176)). Tumor lines were stained for detection of target proteins with the following BioLegend antibodies (CD19 (302212), CD22 (363512), BCMA (357520), SLAMF7 (331810)) and stained with fluorescently tagged isotype controls when indicated. Antigen density was quantified using Quantibrite beads (BD Biosciences, 240495 or custom made). Data were collected on BD FACSymphony A5 and BD FACSCelesta cytometers. FlowJo version #10.8.2 was used to analyze flow cytometry files.

### Generation and fluorescent labeling of extracellular protein domains and density quantification

12X His-tagged CD19 (N138Q) and ICAM-1 extracellular domains were produced using the “Daedalus” mammalian expression system [10]. Briefly, HEK293F cell were transduced with lentivirus expressing CD19(N138Q)-12x His tag or ICAM-1–12x his tag constructs. Proteins were captured from expression culture supernatant by HisTrap FF crude (Cytiva, 11000458) Ni-affinity chromatography and polished by Superdex 200 (Cytiva, 28–9909-44) size exclusion chromatography. Purified proteins were flash frozen in liquid nitrogen in 1X PBS and stored at −80 °C. Extracellular domain of CD22–10x His tag was commercially available (Acrobiosystems, CD2-H52H8). For labeling, 100ug of His-tagged extracellular proteins were concentrated to 1mg/mL using Amicon Ultra Centrifugal Filters (Milipore, UFC500396), and pH adjusted to 8.3 by addition of NaHCO_3_. Proteins were incubated with Alexa Fluor 488 TFP ester, Alexa Fluor 647 NHS ester or Alexa Fluor 555 NHS ester (ICAM_AF488, CD19_AF647, CD22_AF555, CD22_AF647) at room temperature for 15 minutes. Non-reactive dye was removed by size-exclusion chromatography, followed by repeat buffer exchange with Amicon Ultra Centrifugal Filters. Aliquots of protein were frozen and stored at −80° until use. Molecular density of extracellular domains in the soluble lipid bilayer (SLB) was determined using SLB-coated silica beads as previously described [11]. Briefly, silica microspheres (Bangs Laboratories Inc, SS05003) equaling the surface area of a single 96-well chamber were washed and resuspended in PBS. Beads were incubated with lipids (as described in section “soluble lipid bilayer generation and TIRF imaging”) and washed following the same steps as bilayer preparation on 96-well plate. His-tagged proteins were serially diluted and incubated with SLB-coated silica beads for 30 min with gentle shaking. Beads were run on a flow cytometry machine (BD FACSCelesta) along with Quantum Alexa Fluor 488 MESF or Quantum Alexa Fluor 647 MESF (Bangs Laboratories Inc, 647A/488A). Degree of labeling of proteins was determined by 280 and fluorescent absorption, and MESF standards were used to determine the absolute molecular density of proteins on silica beads.

### Soluble lipid bilayer generation and TIRF imaging

Soluble lipid bilayers were generated on 96-well chambered coverslips as previously described [12]. Briefly, 96-well plates (Ibidi, 89627) were washed overnight with 5% Hellmanex III (Sigma, Z805939) and rinsed with ultrapure water. Wells were incubated with 20% HCL 3X, for 1 hr on 50° hot plate. Small unilamellar vesicles (SUV) were generated using 97.5% POPC (Avanti, 850457C-200mg), 0.5% PEG-5000-PE (Avanti, 880230), 2% DOGS-NTA (Avanti, 790404). SUVs were generated by repeated (35X) freeze thaw cycles, moving between liquid nitrogen and a 37° water bath, followed by centrifugation at 33,500×g for 45 minutes at 4°C. SLBs were generated by covering wells with SUV, incubating for 1 hr at 37°, then washed 3X with PBS. SLBs were incubated with his-tagged proteins at determined concentrations (ICAM 200 molecules per μm^2^, CD19 and CD22 50 molecules per μm^2^) for 2 hours, then washed 3X with PBS. T cells were washed and resuspended in cell imaging buffer (RPMI w/o phenol red (Gibco, 11835–03H), 1%FBS, 25mM HEPES), and incubated on bilayer for 40 minutes, followed by fixation with 4% PFA. Bilayers were washed 3X with PBS prior to imaging. Imaging was performed using a Nikon Eclipse Ti2 stand equipped with iLas2 module (Gataca Systems) using the Apo TIRF 100x/1.49 objective and 488nm (515/30), 561nm (595/31) 640nm (860/42) laser lines. Images were acquired with an Andor iXon-L-897 EMCCD camera. The microscope was controlled using Nikon NIS Elements software (version 5.41.01). Distribution of protein within synapse was quantified based on a previously described method [13]. Briefly, radial averages were generated by rotating cell images to all angles 1–359°, all rotated images were compressed to a single stack and z-project of mean intensity was taken. Radial averages combined from all cells measured and intensity values were normalized to max intensity. Analysis was performed in Fiji (ImageJ2, version 2.9.0/1.53t).

### Multiplexed calcium flux measurement by flow cytometry

CAR T cells and ChTCR T cells were harvested and washed once with phosphate buffer saline (PBS). Cells were stained with anti-human CD45 antibody (clone HI30), so that each receptor was stained with a unique CD45 fluorescent barcode (single or double stain with APC-CD45 (Biolegend, 304012), PE-CD45 (BD, 555483), PerCp-Cy-5.5-CD45 (BD, 564105), FITC-CD45 (Biolegend, 304006), BUV805-CD45 (BD, 612891)). Cells were washed three times, pooled 10^7^ cells total. Cells were stained with 5μM indo-1AM dye (Invitrogen, I1223) in calcium stain buffer (phenol-free RPMI, 1%FBS, 0.5 mM probenecid (Sigma, P8761–100G), 10 mM HEPES) at 37° for 45 minutes. Cells were then washed twice with calcium stain buffer, resuspended in 4 mL calcium stain buffer, and split into 4 tubes. Prior to calcium measurement, cells were incubated with biotinylated proteins/antibodies (1 μg/mL CD19-biotin (Accro Biosystems, CD9-H82E9) , 0.5 μg/mL anti-CD28-biotin (Biolegend,302904)) for 5 minutes at 37°. Baseline indo-1AM fluorescence was measured for 30 seconds, before addition of 20μg/mL avidin to crosslink biotinylated proteins. Calcium flux was measured for 5 minutes before addition of 1X cell stimulation cocktail (Invitrogen, 00–4970). Multiplexed populations were deconvoluted and calcium plots generated in FlowJo software (BD), and area under the curve measurements made using Prism software (GraphPad).

### Cell stimulation and western blot

Beads for T cell stimulation were prepared as previously described [14]. 2×10^6^ ChTCR and CAR T cells were washed with resuspended in 50μl warm CTL, incubated with either 30μL/10^6^ beads or an equal number Nalm-6 cells for specified times, immediately washed with 1mL ice-cold PBS and lysed with NP40 RIPA lysis buffer (20nM TRIS pH8, 150mM NaCl, 1% NP40, 5mM EDTA, 0.1%SDS ), supplemented with protease and phosphatase inhibitors (Thermo Scientific, 186093, 78428 ). Cell lysates were sonicated before centrifuging at 10,000g for 15 minutes at 4°C, when present, beads were removed during the lysate clearing step. Total protein concentration was quantified by Micro BCA assay (Thermo Scientific #23235). Equal masses of protein were loaded on Tris-glycine SDS gels (Bio-Rad, 4561086), and proteins were transferred to PDVF membrane (Bio-Rad, 1704274). Membranes were blocked with blocking buffer (Bio-Rad, 12010020), and incubated overnight with primary antibody diluted (1:2000–1:500) in blocking buffer. Membranes were washed three times 5 minutes with Tris-Buffer Saline supplemented with 0.1% Tween, then incubated with secondary antibody diluted in blocking buffer (1:10,000). The following antibodies were used: phospho-LAT (Tyr 220) (Cell Signaling, 3584), phospho-LAT (Tyr 171) (Biolegend, 946602), LAT (E3UCJ) (Cell Signaling, 45533), phospho-Zap 70 (Tyr319) (65E4) (Cell Signaling, 2717), Zap70 (D1C10E) (Cell Signaling, 3165), b-Actin (13E5) (Cell Signaling, 4970) Membranes were incubated with ECL substrate (Bio-Rad, 1705062) and imaged with iBright 1500 Imaging. Band intensities were quantified using ImageJ, normalized to total protein, loading control and control sample as indicated.

### Cytokine measurement

T cell cytokine release was determined by measuring cytokine concentration in the supernatant after 18–24 hours of co-culture of ChTCR and CAR T cells with target cells or with plate-bound antigen. For target cell simulation, T cells and target cells were co-cultured at a 1:1 E:T ratio. After antigen stimulation, supernatant was harvested and cytokine concentration was determined by ELISA according to kit manufacture protocol: IL-2 (BioLegend, 431816) IFN-γ (BioLegend, 430116). For plate-bound antigen stimulation, 96-well plates were coated with avidin (10μg/mL) overnight and incubated with PBS + 3% BSA to block non-specific protein binding. Avidin-coated plates were then coated with biotinylated extracellular protein domains for one hour at specific concentrations. 50,000 T cells were resuspended in 50μL CTL and transferred to antigen coated plates for incubation.

### Cell proliferation assay

ChTCR and CAR T cells were harvested and washed with warm PBS. Cell Trace Violet (CTV) Cell Proliferation dye (Invitrogen, C34557) was resuspended in 200μL DMSO. T cells were resuspended in 1 mL PBS and incubated with 2μL CTV for 10 minutes at 37° C with periodic mixing. 1mL FBS was added to absorbed unbound dye, cells were washed and resuspended in CTL. T cells were co-cultured with Nalm-6 target cells expressing appropriate target antigen at 1:2 E:T ratio for 72 hours. Cells were harvested, washed with PBS, and stained with anti-CD8-APC antibody (Biolegend, 344722) before acquisition by flow cytometry.

### Cytotoxicity assay

Target tumor cells were incubated with Cr^51^ overnight, washed, resuspended in culture media, and plated with effector T cells to achieve indicated E:T ratios. Plates were briefly centrifuged (100rpm for 1 minute), then incubated for 4 hours. After incubation, 30μL of supernatant was harvested, transferred to LumaPlates (Revvity, 6006633), and plates were dried overnight. Plates were read by scintillation counter and percent specific lysis was calculated using the standard formula.

### NSG mouse tumor model

6–8-week-old, female NOD/SCID/γc^−/−^ mice were purchased from Jackson Laboratory or bread in-house. For the Raji model, mice were engrafted with 0.5 million Raji/GFP-ffluc intravenously by tail vain injection. For the Nalm-6 models, mice were engrafted with 0.5 million Nalm-6^WT^ GFP-ffluc or 1 million Nalm-6 GFP-ffluc cells that expressed low levels of CD19 antigens by tail vain injection. For experiments with bi-specific receptors, a heterogenous mixture of Nalm-6^WT^ and Nalm-6 cells expressing only a single antigen were engrafted. Antigen expression in all tumor lines was checked by flow cytometry prior to injection. Mice were injected intracenously 7 days (Raji/GFP-ffluc) or 4 days (Nalm-6/GFP-ffluc) after tumor inoculation with ChTCR of CAR modified CD8^+^ and CD4^+^ T cells at a 1:1 ratio or with PBS. Cell numbers were normalized based on total number of receptor positive cells in the total cell population, as determined by flow cytometry prior to infusion. Mice were followed by bioluminescence imaging after intraperitoneal injection of luciferin substrate using the Xenogen IVIS Imaging System (Caliper Life Sciences) and for survival. Living Image Software V4.7.3 (Caliper Life Sciences) was used to analyze luciferase activity and photon flux within regions of interest that encompassed the entire body of each individual mouse. Blood was obtained from mice at various timepoints, single-cell suspensions from peripheral blood were prepared by lysing red blood cells using ammonium-chloride-potassium (AKC) lysing buffer (Quality Biological, 118–156-101). Single-cell suspensions were stained with the following antibody panel for flow cytometry analysis; Nalm6-GFP, anti-CD45-PE (Biolegend, 304008), anti-CD8-BUV805 (BD, 612889), anti-CD4-cflour R840 (Cytec, R7–20165), rBCMA-biotin (Accro Biosystems, BCA-H82E4), rSLAMF7-biotin (Accro Biosystems, SL7-H82E0), streptavidin-APC (Invitrogen, 17–4317-82).

### Immunoprecipitation of ChTCRs and CARs

HA-tagged ChTCR and CAR lentivirsal constructs generated as described in ‘generation of constructs’ in expressed and expressed in primary human CD8 T cells as described. Cells expressing TCR without HA tag were used as a negative control. 30×10^6^ cells were washed 1X in PBS and lysed in 500uL Co-IP lysis buffer (20mM Tris-HCL pH8, 137mM NaCl, 2mM EDTA. 10% glycerol, 1X protease inhibitor, 1X phosphatase inhibitor (Thermo Scientific, 186093, 78428), 0.5% Brij O10 (Sigma, P6136–100g)), for 30 minutes on ice. Lysate was cleared by centrifugation at 10,00g for 15 minutes at 4°C. 10% of lysate was removed for whole cell lysate controls, and equal masses of the remaining lysate were used for anti-HA immunoprecipitation according to manufacturer instructions (ThermoFisher, 8836). Immunoprecipitated proteins were analyzed by Western Blot as described in “Cell stimulation and Western Blot.” Co-immunoprecipitated proteins were detected using the following antibodies. TCR α (H-1) (SCBT, sc-515719), TCR β (E9I1D) (Cell Signaling, 65123), CD247 (BD Pharmingen, 551034), CD3δ (F-1) (SCBT, sc-137137 HRP), CD3γ (EPR4517) (Abcam, ab134096), CD3ε (D7A6E) (85061), HA-tag (C29F4) (Cell Signaling, 3724)
